# Event- and time-based prospective memory in hemodialysis patients

**DOI:** 10.1080/0886022X.2020.1835673

**Published:** 2020-11-12

**Authors:** Bin Wang, Mengting Li, Fang Tang, Yue Wang, Yuchen Han, Wen Lu, Lan Zhang, Ling Zhang, Weijie Ni, Li Zhang, Liuping Zhang

**Affiliations:** Institute of Nephrology, Zhong Da Hospital, Southeast University School of Medicine, Nanjing, China

**Keywords:** Hemodialysis patients, EBPM, TBPM, cognitive function

## Abstract

**Objective:**

The present study investigated whether hemodialysis (HD) patients exhibit future memory impairment (PM; the capability of remembering to perform expected future actions) and exploring relevant factors of PM task performance.

**Methods:**

Sixty HD patients and 60 healthy controls matched by age are enrolled in the Mini-Mental State Examination (MMSE), Finger Span Test (DST), Oral Fluency Test (VFT), Ray Auditory Oral Learning Test (RAVLT), Received Stroop Color Word Interference Test (SCWT), and event-based PM (EBPM) and time-based PM (TBPM).

**Results:**

There were no significant difference between the patients and controls in the DST-Forward digit span (9.00 ± 1.25 versus 8.97 ± 1.33, *p* = 0.96), the DST-Backward digit span (5.23 ± 1.98 versus. 4.60 ± 1.65, *p* = 0.11), the RAVLT of delayed recall (7.28 ± 2.36 versus 6.87 ± 3.33, *p* = 0.09) and the VFT for animals (16.70 ± 3.50 versus 17.68 ± 5.45, *p* = 0.56). By comparison, patients had a much worse performance than controls on the MMSE (29.10 ± 0.84 versus 28.33 ± 0.77, *p* < 0.001), the RAVLT of total recall (44.47 ± 5.82 versus 40.03 ± 10.46, *p* < 0.001) and delayed recognition (6.93 ± 1.49 versus 5.4 ± 1.33, *p* < 0.001), the SCWT reaction time in reading (6.47 ± 1.05 versus 7.47 ± 1.86, *p* < 0.001), color naming (9.07 ± 1.29 versus 11.43 ± 2.34, *p* < 0.001), interference (8.78 ± 1.92 versus 10.22 ± 2.91, *p* < 0.001) and inhibition/switching (14.53 ± 2.90 versus 19.85 ± 4.69, *p* < 0.001), the VFT for fruit (17.47 ± 3.18 versus 15.92 ± 4.56, *p* < 0.001), the EBPM task (7.85 ± 0.40 versus 7.08 ± 1.43, *p* = 0.01), and the TBPM task (3.30 ± 1.31 versus 2.26 ± 1.82, *p* < 0.001).

**Conclusions:**

Our results suggest that EBPM and TBPM are impaired in HD patients and that PM may be applied to help evaluate cognitive dysfunction in HD patients.

## Introduction

Cognitive impairment is one of the most characteristic symptoms of uremic encephalopathy [1] and also manifests commonly among people with end-stage renal disease (ESRD). 90 and 77% of hemodialysis (HD) patients are reported with modest and severe cognitive complaints in memory and other cognitive domains, respectively [2]. It is also demonstrated the annual rate of severe dementia among old patients was 7.4 times higher in hemodialysis patients than that of most people [3]. There is also a proven correlation between cognitive impairment and a growing risk of mortality [4]. Even if the patients are fully dialyzed, some of them are still suffered from uremic encephalopathy or other signs of nervous system disorders. Most studies investigating cognitive problems in uremic patients have focused on, attention, global cognitive and executive function, and memory retardation [5]. However, whether the uremic patients had a positive memory (PM) problems remains unknown.

PM is defined as a memory of future actions [6]. It also has a strong relationship to routine business including keeping in mind the time of a day to take medicine and to pick up the kids from school [7]. Since PM is a cognitive process that is closely related to daily affairs, the reduction of it may cause negative results of professional, social, or health-related issues [8]. PM can be subdivided into two categories: event-based PM (EBPM) and time-based PM (TBPM). The former refers to remembers to engage in action triggered by exogenous causes such as not forget to inform a friend of an appointment. The latter is described to remember the fixed-time action, including the necessity of taking prescribed medication at 8 am. At present, several published training methods may help adults with impairment of PM [9,10].

It is significant for HD patients to perform intact PM to successfully accomplish daily routines. The present study is to demonstrate whether HD patients have PM dysfunction as well as verify the influencing determinants.

## Methods

### Participants

**Figure 1.**
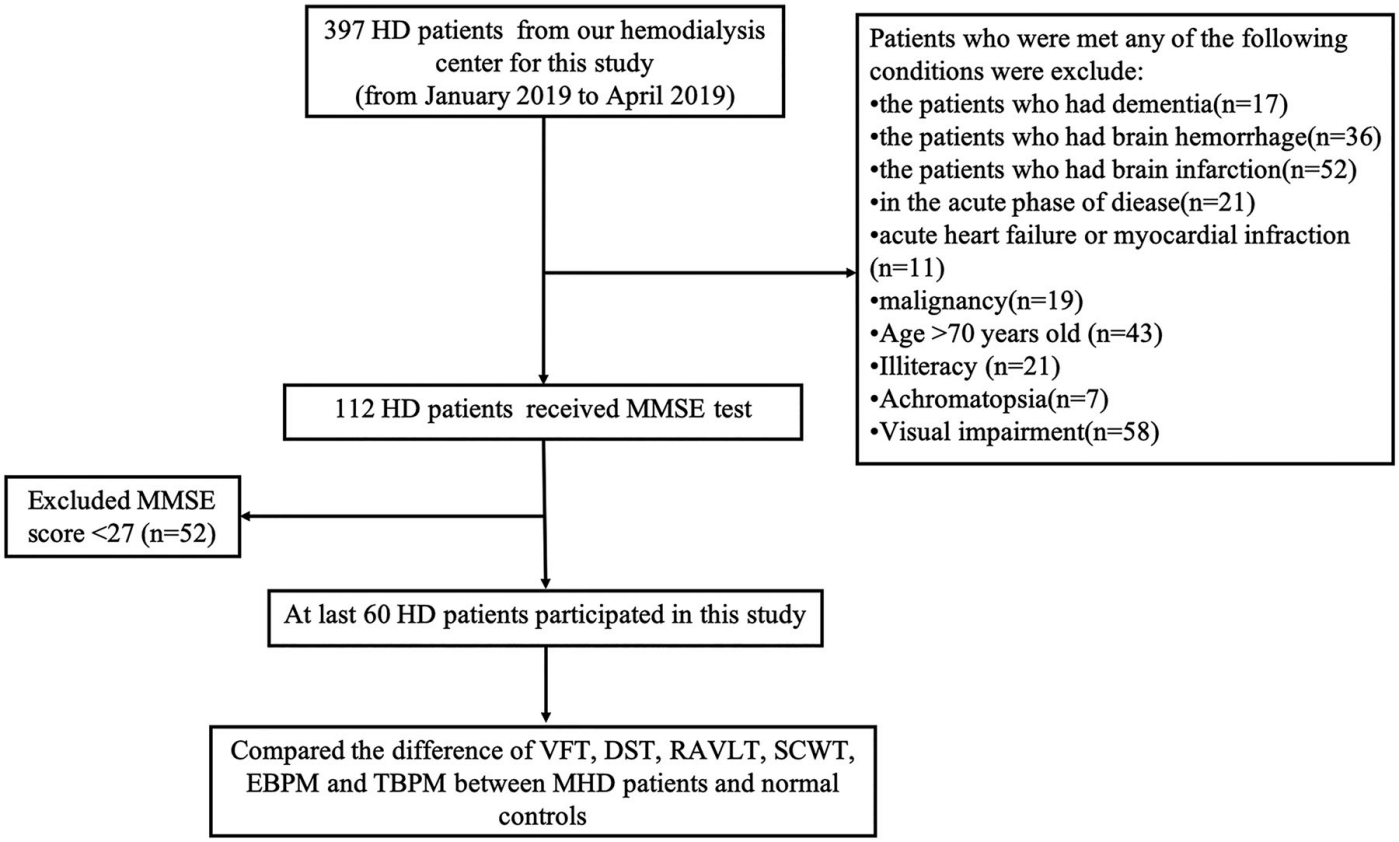
Participant flow diagram.

The cohort is consists of 60 HD patients (Figure 1) and 60 controls matched by age and all of them gave informed consent. All of the 60 HD patients were outpatients at the hemodialysis center of the Zhongda hospital of Southeast University (mean age = 45.55 years, SD = 12.35), and 60 controls were local healthy residents (mean age =44.83 years, SD= 13.00). Inclusion criteria: HD patients who undergo hemodialysis 2 or 3 times a week, URR > 65%; age ≤70-years-old; without a history of other diseases or disorders in the nervous system, and the Mini-Mental State Examination (MMSE) was scored 27 of 30 or above; have the ability to take communication in Chinese and understand the meaning of questionnaire; gave informed consent and are volunteer to participate in this study. Exclusion criteria: cerebral infarction or/and hemorrhage, dementia, acute heart failure, or other acute diseases, achromatopsy or/and visual disability. The mean span of hemodialysis was 7.3 years with a range from 1 to 24 years. The persons in the control group were matched to the HD patient group in terms of sex, age, and socioeconomic status. HD patients were examined between January and March 2019, and the control participants were examined between February and April 2019. The study protocol was approved by the Ethics Committee of Zhongda Hospital (2018ZDKYSB167).

### Neuropsychological evaluation

Several tests were conducted to evaluate general memory and cognitive functions: the MMSE [11] was for cognitive impairment; the Verbal Fluency Test (VFT) [12] was to assess frontal lobe functions, Subjects were required to express words of animals and vegetables in Chinese as many as possible within one minute.; the digit span test (DST) [13] was for measurement of the attention and span of memory; the Rey Auditory Verbal Learning Test (RAVLT) [14] was for assessment of verbal memory, new learning, susceptibility to intervention, and recognition memory, and the modified Stroop Color Word Interference Test (SCWT) [15] was for measurement of the selective attention [16].

### Event-based prospective memory task

Participants started by practicing the ongoing tasks of working memory first, which required participants to remember eight target words belonging to the animal within 30 s [16,17]. The PM guidance was introduced for the succession of the practice trials for the ongoing task. 32 question cards were shown to participants and 12 words in common Chinese were printed on each card, ten of which referred to one classification (vegetables) with the remaining two words were of another type (animals). Subjects should choose two words of one classification that differed from the other 10 words and they also needed to complete the task of selecting the target words hidden in the mission. Participants should tap the desk when the elected Chinese word represented target words during the subsequent tasks. The target words for the PM task appeared on the rounds of 2nd (camel, rat), the 7th (monkey, snake), 13th (camel, elephant), 20th (cat, dog), 24th (elephant, monkey), and 29th (snake, bear). The participants were required to recall the names of target words and give a contact number when they finishing the word selection task.

The recorded PM score reflected the performance of the participant on the PM task. The participants who remembered to give a contact number scored 2 scores after the word selection task. Each correct response to a target word was given a score of 1, and those who do not answer correctly are not scored (maximum score 8). The RM score was used to express the subject’s responses to recalling the names of all the animals after the word selection task. One score point for every correct answer to recalling of an animal (maximum score 6). The time for administration of the EBPM was obtained.

### Time-based prospective memory task

For the PM task, the subject was initially told to tap the desk in 5, 10, or 15 min from the start time of the task [16,17]. 100 question cards with 12 two-digit numbers on each card were displayed for the subjects to figure out the smallest number of the first 10 cards and the largest number of the following 10 cards. A digital clock was set approximately one meter away behind the subjects in order to check the time at any time. The subjects need to turn behind to check the clock, and every turn was recorded by the experimenter. The subjects were allowed to perform the number selection task when the clock started and to stop the number selection task within the time limit of 17 min. The frequency of the participant checked the clock per minute was recorded and the subjects were asked to report the number of times of tapping the desk.

Referring to the PM task, participants who responded between the first 10 s and the last 10 s of the target time were counted as 2 points, and the remaining subjects were counted as 0 marks; (maximum score 6). Referring to RM, if the time that the subjects recalled the time point of tapping the table was correct, then 2 points were given (maximum score 6). Of both groups, 10 subjects started with the EBPM task while the other 10 subjects started with the TBPM task.

### Statistical analysis

The data that followed a normal distribution were described by the mean (SD) and were conducted using an independent *t*-test; while the median (interquartile range (IQR)) and the Mann–Whitney *U* test were used. The proportions variables were analyzed by Chi-square test. The correlations of PM performance, demographic, clinical characteristics, and other neurocognitive functions were analyzed by Pearson or Spearman correlation test. Significance was defined by a *p*-value < 0.05. The statistical analyses were performed by SPSS 25.0 software.

## Results

### Neuropsychological evaluation

The proportion of hypertensive patients in HD patient group is higher than that of the control group (*p* < 0.001). In addition, HD patients performed equally in the DST forward digital span test and the DST backward digital span test (*p* > 0.05) in comparison with the control group. However, compared to HD patients, the control group showed significantly better MMSE (*p* < 0.01), better RAVLT, but only in the total recall and delayed recognition (*p* < 0.01), better VFT performance on fruit (*p* < 0.01), and better SCWT reaction time (*p* < 0.01). Table 1 is a summary of the results of the neuropsychological tests.

**Table 1.** The demographic data and neuropsychological test results for the HD patients and controls.

	Controls (mean ± SD)	HD patients (mean ± SD)	*p*-Value
Age, years	44.83 ± 13.00	45.55 ± 12.35	0.76
Education, years	13.28 ± 2.15	13.05 ± 1.87	0.61
Male (%)	34 (56.7%)	34 (56.7%)	1.00
Duration of hemodialysis, years		7.3 ± 5.0	
Hypertension (%)	23 (38.3%)	47 (78.3%)	<0.001^a^
Diabetic (%)	5 (8.3%)	10 (16.7%)	0.17
MMSE	29.10 ± 0.84	28.33 ± 0.77	<0.001^a^
DST-Forward digit span	9.00 ± 1.25	8.97 ± 1.33	0.96
DST-Backward digit span	5.23 ± 1.89	4.60 ± 1.54	0.11
RAVLT			
Total recall	44.47 ± 5.82	40.03 ± 10.46	<0.001^a^
Delayed recall	7.28 ± 2.36	6.87 ± 3.33	0.09
Delayed recognition	6.93 ± 1.49	5.40 ± 1.33	<0.001^a^
SCWT			
Reaction time (s)			
Reading	6.47 ± 1.05	7.47 ± 1.86	<0.001^a^
Colors naming	9.07 ± 1.29	11.43 ± 2.34	<0.001^a^
Interference	8.78 ± 1.92	10.22 ± 2.91	<0.001^a^
Inhibition/switching	14.53 ± 2.90	19.85 ± 4.69	<0.001^a^
Error			
Reading	0.00 ± 0.00	0.03 ± 0.18	0.16
Colors naming	0.00 ± 0.00	0.10 ± 0.35	0.02
Interference	0.03 ± 0.18	0.08 ± 0.28	0.25
Inhibition/switching	0.08 ± 0.28	0.35 ± 0.63	<0.001^a^
VFT (animal)	16.70 ± 3.50	17.68 ± 5.45	0.56
VFT (fruit)	17.47 ± 3.18	15.92 ± 4.56	<0.001^a^

^a^Mann–Whitney *U* test.

MMSE: Mini-Mental State Examination; DST: the digit span test; RAVLT: Rey Auditory Verbal Learning Test; SCWT: Stroop Color Word Interference Test; VFT: Verbal Fluency Test.

**Table 2.** Performance of the control group and the HD patients on subitems of the event based prospective memory tasks.

	Controls (mean ± SD)	HD patients (mean ± SD)	*p*-Value
Prospective memory score	7.85 ± 0.40	7.08 ± 1.43	0.001*
Retrospective memory score	4.75 ± 1.34	3.37 ± 1.47	<0.001*

*Mann-Whitney U test.

**Table 3.** Performance of the control group and HD patients on subitems of the TBPM tasks.

	Controls (mean ± SD)	HD patients (mean ± SD)	*p*-Value
Prospective memory score	3.30 ± 1.31	2.26 ± 1.82	<0.001^a^
Retrospective memory score	3.23 ± 1.43	2.37 ± 1.54	0.002^a^
Number of clock checking responses	32.33 ± 2.32	27.88 ± 3.95	<0.001^a^

^a^Mann–Whitney *U* test.

### EBPM

The EBPM task showed that the PM and RM scores of the HD patient group (7.08 ± 1.43 and 3.37 ± 1.47) were significantly lower than those of the control group (7.85 ± 0.40 and 4.75 ± 1.34) (*p* < 0.01), indicating that EBPM was impaired in the HD patient group (Table 2).

### TBPM

**Figure 2.**
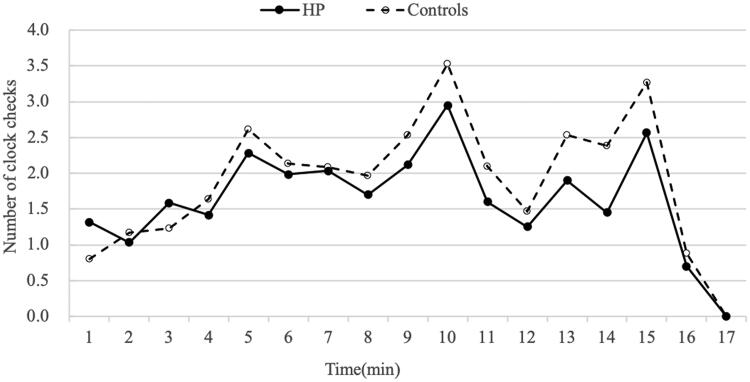
Mean number of clock checking responses in the control group and the HD patients group in each one minute period during the EBPM task.

The TBPM task and RM task showed that the HD patients’ scores (PM 2.26 ± 1.82; RM 2.37 ± 1.54) were lower than those of the controls (PM 3.30 ± 1.31; RM 3.23 ± 1.43) (all *p* < 0.01) (Table 3). To examine whether performance on the TBPM task was related to the frequency with which HD patients monitored the time compared with controls, the mean number of overall clock-checking responses was calculated and is displayed in Figure 2. The frequency of clock checking in the HD patient group (27.88 ± 3.95) was significantly lower than that in the control group (32.33 ± 2.32) (*p* < 0.01) (Table 3).

### Factors correlated with PM task performance

Correlation analyses were performed for the EBPM and TBPM scores with demographics, clinical variables and neuropsychological parameters, listed in Table 1, among the HD patients. Only the DST-Forward digit span and DST-Backward digit span were significantly correlated with the EBPM score (*p* < 0.05). TBPM was correlated with the duration of hemodialysis, RAVLT-Total recall, RAVLT-Delayed recall, SCWT-Reading-Error and SCWT-Inhibition/switching-Error (Table 4, all *p* < 0.05).

**Table 4.** Correlation analyses for the EBPM and TBPM score with demographics, clinical variables and neuropsychological parameters in HD patients.

	PM tasks			
	EBPM		TBPM	
	*r*	*p*	*r*	*p*
Age	−0.0182	0.163	−0.038	0.771
Education	0.087	0.508	−0.123	0.348
Duration of hemodialysis	0.037	0.779	0.323	0.012*
MMSE	0.174	0.185	0.176	0.178
DST-Forward digit span	0.386	0.002*	0.200	0.125
DST-Backward digit span	−0.138	0.292	0.376	0.003*
RAVLT-Total recall	0.331	0.010*	0.238	0.067
RAVLT-Delayed recall	0.125	0.342	0.263	0.042*
RAVLT-Delayed recognition	0.124	0.347	0.325	0.011*
SCWT-Reading-Time	−0.352	0.006*	−0.057	0.664
SCWT-Colors naming-Time	−0.340	0.008*	0.028	0.831
SCWT-Interference-Time	−0.143	0.276	0.072	0.585
SCWT-Inhibition/switching-Time	−0.251	0.054	−0.019	0.883
SCWT-Reading-Error	0.120	0.361	0.178	0.173
SCWT-Colors Naming-Error	−0.251	0.053	−0.042	0.750
SCWT-Interference-Error	−0.230	0.077	−0.111	0.397
SCWT-Inhibition/switching-Error	−0.145	0.269	0.123	0.347
VFT (animal)	0.425	0.001*	0.394	0.002*
VFT(fruit)	0.323	0.012*	0.313	0.015*

EBPM: event based prospective memory; TBPM: time based prospective memory; MMSE: Mini-Mental State Examination; DST: the digit span test; RAVLT: Rey Auditory Verbal Learning Test; SCWT: Stroop Color Word Interference Test; VFT: Verbal Fluency Test.

*p value < 0.05.

## Discussion

Chronic renal failure (CRF) and dialysis treatment have been confirmed to affect the central nervous system and neurocognitive function [18]. PM is collated with various areas of the brain as a significant component of memory [19]. In addition, compared to RM, PM is explained more in terms of day-to-day memory functioning, which is required in self-care, self-management and monitoring. Previous studies have indicated that there may be a tendency for HD patients to perform more poorly in laboratory-based cognitive tasks. However, little prior research has evaluated the status of PM in HD patients. Thus, we focused on the condition of PM in HD patients to understand the relationship between HD and cognitive function. Our results showed significant impairments of PM (both EBPM and TBPM) in HD patients. This PM impairment is related to the duration of hemodialysis.

The need for research into the PM problems of HD patients is not only of diagnostic importance but also of therapeutic significance. Because of the absence of objective measures of early uremic encephalopathy, the detection of PM impairment may be used as a foundation for diagnosing early uremic encephalopathy. In our study, some of the included HD patients were undergoing an impaired PM, although they performed normally in the MMSE. In this study, some HD patients with impaired PM, performed normally in the MMSE. indicating that PM tasks may be more effective for screening cognitive impairment in HD patients. Besides, it is significant for HD patients to be restricted of fluid restrictions and compliant with the guidelines of medication and diet [20]. Partially due to declining memory function, non-adherence to prescribed drugs significantly increases morbidity and mortality in developing countries in CKD patients [21]. Therefore, HD patients may be given additional help to manage their PM difficulties. Due to PM difficulties in HD patients, family and multidisciplinary team support may be helpful for improving their treatment adherence.

As far as we know, this is the first study to evaluate PM impairment in HD patients. PM has recently been used as an evaluation indicator in many clinical studies of diseases, such as Parkinson’s and Alzheimer’s disease, which were with PM impairment [22,23]. The spontaneous extraction of PM information was related to the functions of the prefrontal cortex, and there were significant PM impairment in patients with frontal lobe impairment [24,25]. While both EBPM and TBPM are controlled by the prefrontal cortex, patients with lesions in the prefrontal cortex exhibit impairments in EBPM and/or TBPM [21]. The morphological characteristics of the pyramidal neurons of the medial part of the prefrontal cortex are changed by the chronic effect of high blood pressure, which is very common in ESRD [26]. Furthermore, impairment of prefrontal cortex functions has been found in predialytic chronic kidney disease (PDCKD) patients and HD patients [27] and is explained in terms of neuropathological impairment in the prefrontal area. Additionally, Fazekas G et al. found that region over cerebellar activity was significantly reduced in the frontal cortex and thalamus of the HD patients [28]. Our study found that the TBPM is heavily dependent on time-monitoring behaviors (such as clock checking), which is believed to be dependent upon the activation of thalamus [29–31]. Hence, the impaired prospective component of PM may due to the prefrontal cortex and thalamus dysfunction in the HD patients.

Many clinical characteristics could also be used to explain the observation of PM deficits among HD patients. First, PM deficits may be related to the high percentage of cerebral atrophy (CA) found in HD patients [32]. Tenkku M et al. [33] demonstrated that patients with central cerebral atrophy had poor memory performance. An alternative explanation may be related to the fact that conventional hemodialysis can cause recurrent acute cerebral ischemia [27]. Mark et al. showed that patients undergoing hemodialysis experience transient declines in cerebral blood flow, correlating with intradialytic cognitive dysfunction [34], which in turn may contribute to a chronic decline in PM function. Additionally, small-vessel cerebrovascular disease is the most common cause of vascular dementia [35]. HD patients often have a series of recognized accelerators of vascular damage risk factors, including hyperlipidemia, hypertension, and an elevated inflammatory state. Finally, the accumulation of various uremic toxins caused by renal failure [36], which plays an important role in the etiology of uremic encephalopathy, might be responsible for the impairment of PM.

This study presents some limitations. First, this study used a simple PM task to evaluate instead of imaging examinations, which is very preliminary. However, the results were inspiring. We hope that more complex PM tasks combined with fMRI could be applied to investigate the neural mechanisms in HD patients in the future. Second, this was a cross-sectional study. It is hard to exclude prehemodialysis cognitive deficits in HD patients, which may have influenced the results. Third, the sample is small, and the scoring system has a limited variation of scores, thus, rendering the statistical analysis difficult. Last but not least, there are some possible biases, including admission rate bias and volunteer bias: for example, the HD patient group had more hypertensive patients than the control group. Therefore, the results of the current study should be interpreted with caution, and large-sample and multicenter studies are needed to further support the results of our study in the future.

In conclusion, our study confirmed that EBPM and TBPM were impaired in HD patients, indicating that PM evaluation may be useful for screening the cognitive dysfunction and improving the treatment compliance and prognosis of HD patients.
